# Case Report: Novel *MAGT1* pathogenic variant with significant atopy, hypogammaglobulinemia and viral skin infections

**DOI:** 10.3389/fimmu.2026.1754394

**Published:** 2026-02-16

**Authors:** Lauren Gunderman, Christopher P. Ptak, Madeline Schutt, Kento Yahashiri, Elizabeth Lippner, Amer Khojah, Aisha Ahmed, Aaruni Khanolkar

**Affiliations:** 1 Division of Allergy and Immunology, Ann & Robert H. Lurie Children’s Hospital of Chicago, Chicago, IL, United States; 2 Department of Pediatrics, Northwestern University Feinberg School of Medicine, Chicago, IL, United States; 3 Department of Pediatrics, University of Washington School of Medicine, Seattle, WA, United States; 4 Division of Immunology, Seattle Children’s Hospital, Seattle, WA, United States; 5 Biomolecular Nuclear Magnetic Resonance Facility, University of Iowa, Iowa City, IA, United States; 6 Department of Molecular Physiology and Biophysics, Carver College of Medicine, University of Iowa, Iowa City, IA, United States; 7 Biological Sciences, Northwestern University, Evanston, IL, United States; 8 College of Medicine, Umm Al-Qura University, Makkah, Saudi Arabia; 9 Department of Pathology, University of Iowa, Carver College of Medicine, University of Iowa, Iowa City, IA, United States

**Keywords:** AlphaFold, atopy, congenital disorders of glycosylation (CDG), inborn error of immunity (IEI), MAGT1, NKG2D (Natural killer group 2 member D), XMEN disease, Oligosaccharyltransferase-B (OST-B)

## Abstract

**Background:**

X-linked MAGT1 deficiency with increased susceptibility to EBV-infection and N-linked glycosylation (XMEN) disease is an inborn error of immunity (IEI) affecting the Magnesium Transporter 1 (*MAGT1)* gene. In this report, we present the diagnostic odyssey for a patient harboring a novel *MAGT1* variant resulting in XMEN disease.

**Case presentation:**

A 6y old male child of Caucasian ancestry presented at the immunology clinic in our hospital with a history of recurrent upper respiratory tract infections, as well as significant atopy and viral skin lesions. Genetic testing identified a novel, hemizygous pathogenic variant in the magnesium transporter 1 (*MAGT1*) gene (c.580dup; p.Ser194Phefs*3). Follow-up testing by flow cytometry revealed the canonical disruption in Natural Killer Group 2D (NKG2D) surface expression on CD8 T cells and NK cells, and clinical testing for congenital disorders of glycosylation (CDG) additionally verified the hallmark defect in glycosylation that underpins XMEN disease. Subsequent *in silico* analyses using AlphaFold provided an in-depth view of the resulting aberrant protein structural variant and its inability to tether itself to the OST-B complex, a pre-requisite for optimal enzymatic activity of the MAGT1 protein. Disease management included infection control and prophylaxis, steroids and immunotherapy for the patient’s asthma and atopy, topical antiviral treatment for the warts and molluscum, as well as biannual EBV load monitoring (the patient is EBV negative).

**Conclusion:**

This case illustrates how a synergistic multi-disciplinary team approach established a diagnosis of XMEN disease in a patient with an atypical clinical presentation. This case also highlights a growing trend where established diagnostic tools such as flow-cytometry and genomics can be complemented with newer, sophisticated analytical approaches such as AlphaFold to further elucidate the functionally crippling effects of novel variants described in the setting of IEI.

## Introduction

Mutations in the highly conserved magnesium transporter 1 gene (*MAGT1*) were identified in 2011 and described in patients with an X-linked inborn error of immunity (IEI) characterized by CD4+ T cell lymphopenia, severe chronic viral infections, and defective T lymphocyte activation (1). Initially, the compromised immunity observed in MAGT1 deficiency was attributed to its potential role as a mediator of Mg^2^+ influx into T cells, which helps regulate the Ca^2+^ influx dependent signaling cascade required for optimal activation of T cells (2). MAGT1 was found to be a component of the oligosaccharyl transferase (OST) complex in the endoplasmic reticulum, participating in N-linked glycosylation (NLG) of substrate proteins, and follow up studies established that it’s the aberrant glycosylation that primarily underpins the resulting immune defects that accompany loss of MAGT1 expression and/or function (3). One of the substrate proteins whose optimal surface expression relies on MAGT1-induced N-linked glycosylation is Natural Killer Group 2 member D (NKG2D), a key effector molecule expressed by cytotoxic CD8 T cells and NK cells that play a critical role in EBV immunosurveillance (3). EBV+ lymphoblastoid cells express high levels of ligand molecules that engage NKG2D. Decreased expression of NKG2D on the surface of NK cells and CD8 T cells in the setting of MAGT1 deficiency affects their ability to eliminate EBV-infected cells and consequently increases the likelihood of developing EBV-associated malignancies (3, 4). The clinical spectrum of MAG-T1 deficiency includes recurrent sinopulmonary infections, lymphadenopathy, bronchiectasis, hypogammaglobulinemia, antibody-mediated cytopenias, lymphomas, persistent molluscum and, less frequently, neurological abnormalities (3). Here, we describe an unexpected diagnosis of MAGT1 deficiency in a patient presenting prior to EBV infection with initial symptoms of significant atopy, recurrent infections and molluscum contagiosum, followed by in-depth structure and function modeling analyses of the affected MAG-T1 protein using AlphaFold (5).

## Methods

### Flow cytometry

Circulating lymphocytes in whole blood samples from the patient and healthy controls were immunophenotyped using multi-parameter flow cytometry. Hierarchical gating and lineage-specific markers were utilized to identify T cell subsets, B cells and NK cells. NKG2D, TCRαβ and CD5 surface expression was evaluated using the following antibodies: mouse anti-human NKG2D Ab (clone 1D11; Biolegend, San Diego, CA), mouse anti-human CD5 Ab (clone L17F12; BD Biosciences, San Jose, CA), and mouse anti-human TCRαβ (clone WT31; BD Biosciences, San Jose, CA). The following isotype control antibodies were included to determine the level of background staining for NKG2D, CD5 and TCRαβ expression: mouse IgG1, κ (clone MOPC-21, Biolegend, San Diego, CA), mouse IgG2a, κ (clone MOPC-173, Biolegend, San Diego, CA) and mouse IgG1, κ (clone X40, BD Biosciences, San Jose, CA). Marker-directed and isotype control antibodies were used at matching doses. A total of 100,000-200,000 events were acquired for each sample using a FACS Canto II flow-cytometer (BD Biosciences, Franklin Lakes, NJ), and data were analyzed using FlowJo software (version 10.8) (BD Biosciences, Franklin Lakes, NJ).

### Case description

**Figure 1:**
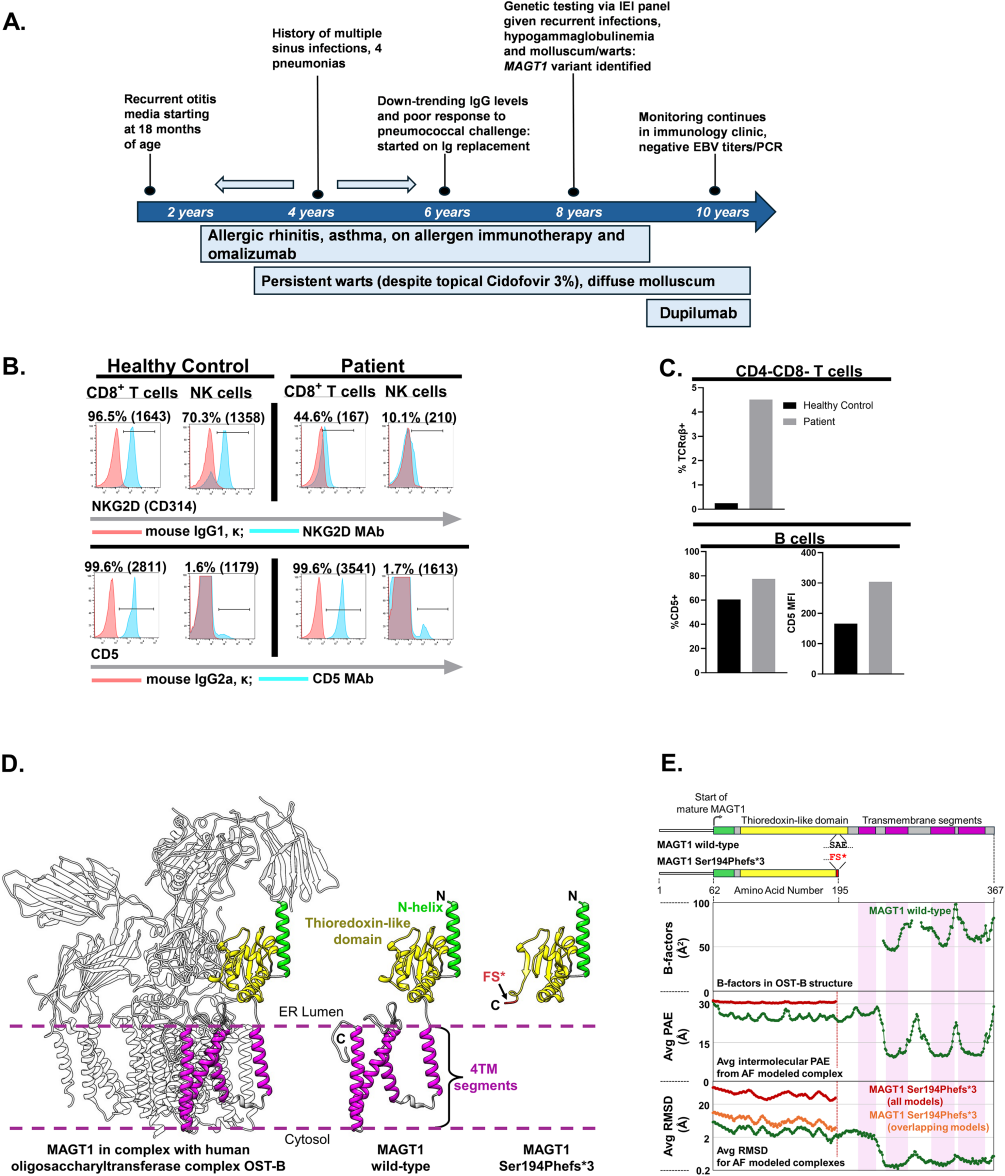
**(A)** Schematic depicting the timeline of the clinical presenting features of the patient **(B)** Evaluation of NKG2D and CD5 surface expression. Polychromatic flow cytometry was utilized to measure surface expression of NKG2D as a surrogate readout of aberrant glycosylation. The numerical values listed on top of each plot indicate the background-subtracted frequencies (%) of CD8 T cells and NK cells expressing NKG2D and CD5 on the surface. The adjacent numerical values listed in parentheses represent the background-subtracted median fluorescence intensities (MFI) of NKG2D (top row) and CD5 (bottom row) expression. **(C)** Analysis of TCRαβ^+^ double negative (CD4^-^CD8^-^) T cells (top-row) and CD5^+^ B cells (bottom row). The expression of surface TCRαβ on gated CD3^+^CD4^-^CD8^-^ T cells and CD5 on the surface of B cells was examined by flow cytometry. The top bar-graph represents the background-subtracted frequencies (%) of CD3^+^CD4^-^CD8^-^ T cells expressing TCRαβ (Reference range: <2.6% of CD3^+^CD4^-^CD8^-^ T cells express TCRαβ). The bottom bar-graph represents the background-subtracted frequencies (%) CD5^+^B cells and the MFI of CD5 expression on the surface of B cells. Samples were acquired on FACS Canto-II flow cytometers (Becton Dickinson, Franklin Lakes, NJ, USA), and the flow cytometric data were analyzed using FlowJo software (version 10.8.0)(Ashland, OR, USA). **(D)** Models for the mature MAGT1 wild-type protein (center) and MAGT1 Ser194Phefs*3 protein (right) were generated with AlphaFold3. The thioredoxin-like domain (yellow) is positioned in the ER Lumen, while the 4 transmembrane (TM) segments (magenta) are embedded in a lipid bilayer. For the variant Ser194Phefs*3, the amino acid changes linked to the frameshift are indicated in red. The MAGT1 wild-type model was aligned with the resolved MAGT1 segments within the OST-B structure (PDBid: 6S7T) (white) (left). **(E)** 1D schematic representations of wild-type and Ser194Phefs*3 proteins are colored and scaled to match the amino acid numbers in the structural models. Numbering corresponds to starting and ending positions for each protein. An amino acid alignment (inset between 1D representations) details the differences between wild-type and Ser194Phefs*3 for positions 194-196. B-factors for MAGT1 amino acids in the cryoEM structure indicate positional uncertainty for regions of modeled density (top plot). AlphaFold3 co-complexes of STT3B and each MAGT1 protein were modeled and analyzed for prediction confidence and model variability. Intermolecular Predicted Aligned Error (PAE) values for the position of MAGT1 amino acids relative to co-modeled STT3B were averaged to generate a single value at each MAGT1 amino acid. For the 5 AlphaFold3 models, PAE values were averaged and plotted to show regions of increased confidence (< 15 Å) (middle plot). MAGT1 wild-type (green) and Ser194Phefs*3 (red) are plotted together. The α-carbon RMSDs for MAGT1 models were calculated against the top-ranking predicted structure after alignment of the co-complex on STT3B. For each MAGT1 protein, the 4 RMSD data sets were averaged and graphed on a log scale (bottom plot). While all 5 MAGT1 wild-type models overlaid, only 3 MAGT1 Ser194Phefs*3 docked to a similar position relative to STT3B. A second plot of RMSD for only overlapping Ser194Phefs*3 models (removing the 2 distinct docking poses) is included (orange). For each plot, transmembrane segments are indicated by shaded boxes (light magenta) and TM segments 2–4 correlate with the resolved cryoEM density regions as well as higher confidence in positioning and lower structural variability with respect to STT3B.

The patient is a male child who presented at the age of 6y (**Figure 1A**). The patient was healthy until 18 months of age when he began developing recurrent otitis media. He then developed four episodes of pneumonia and multiple sinus infections. He also had moderate atopic dermatitis, a chronic cough and was under the care of an allergist for management of his severe persistent asthma and allergic rhinitis. On initial examination, the patient was observed to have short stature and persistent warts on bilateral hands as well as persistent, widespread molluscum contagiosum lesions. Family history was pertinent for mother with chronic rhinosinusitis and maternal grandfather diagnosed with asthma, atopic dermatitis and chronic sinus issues. Initial immunology laboratory assessment revealed a serum IgG level of 524 mg/dL [age-associated reference range (RR): 608–1229 mg/dL)], and a serum IgA level of 20.8 mg/dL [RR: 33–200 mg/dL], and subsequent assessments revealed a progressive decline in IgG levels (**Table 1**). In contrast, initial serum IgM levels were within normal limits [56.6 mg/dL (RR: 46–197 mg/dL)] and serum IgE was slightly elevated [137 KU/L (RR: <126 KU/L)]. Longitudinal assessment of serum IgE revealed it to be persistently elevated with a peak of 1173 KU/L (RR: < 176 KU/L). Response to the Streptococcus pneumoniae unconjugated, pure polysaccharide vaccine evaluated at the time of initial presentation was protective for only 12 out of the 23 serotypes (>1.3 μg/mL) (RR: ≥ 1.0 μg/mL for >70% of the serotypes), while the Tetanus-specific IgG level was protective (>0.15 IU/mL) (6, 7). Of note, CD3, CD4 and CD8 T cell, NK cell and B cell absolute counts were within the normal ranges. Collectively, these findings suggested a Common Variable Immunodeficiency (CVID)-like phenotype, although the proportion of isotype-switched memory B cells was within normal limits (**Table 2**) (8, 9).

**Table 1:** Serum IgG levels (prior to initiating Ig replacement therapy).

Patient age	Serum IgG (mg/dL)[reference interval for age range of 6y-8y: (608-1229)]
6y9m	524
7y	485
7y3m	498

y, years; m, months.

**Table 2:** Circulating Naïve and Memory B cell subset analysis (patient age: 7y7m).

Subset*	Patient value (% of CD19+ B cells)	Reference Range (% of CD19+ B cells) (Age-group: >5y)
Naïve B cells (CD19+IgD+CD27-)	82.87	54.85-96.02
Isotype-Unswitched Memory B cells CD19+IgD+CD27+	4.71	0.65-11.96
Isotype-Switched Memory B cells CD19+IgD-CD27+	8.33	1.14-26.87
Double-Negative Memory B cells CD19+IgD-CD27-	4.09	1.21-12.61
Plasmablasts CD19+CD20-CD38+CD27+	0.28	0.00-4.23
Transitional B cell subsets (CD19+CD24hiCD38hi)		
CD10+CD21-	0.02	0.00-0.04
CD10+CD21+	0.02	0.00-0.32
CD10-CD21+	2.00	0.12-8.64
CD10-CD21-	0.46	0.00-0.12

*Subset definitions adapted from Maecker, H.T. et al, Nat Rev Immunol, 2012, 12(3):191-200, Suryani, S. et al, Blood, 2010;115(3):519-29, and Wei, C, et al, J Immunol. 2007;178(10):6624-33 (38–40).

y, years; m, months.

Genetic testing revealed a novel hemizygous pathogenic variant in the magnesium transporter 1 (*MAGT1*) gene (c.580dup; p.Ser194Phefs*3) which creates a duplication at nucleotide position 580 generating a premature stop codon three amino acids downstream from the variant locus, resulting in an absent or abnormal protein product (loss of function). A second variant in the patient’s *MAGT1* gene (c.574G>A; p.G192S) was identified as a variant of unknown significance. Testing for Congenital Disorders of Glycosylation (CDG) revealed moderately elevated transferrin mono-oligosaccharide to di-oligosaccharide, and Apo CIII-1/Apo CIII-2 ratios, consistent with the diagnosis of CDG (data not shown).

With family consent, testing was pursued to examine surface NKG2D expression on CD8+ T cells and NK cells by flow cytometry as the abrogation of NKG2D expression is currently the best diagnostic biomarker of the disease (**Figure 1B**; upper row). The median fluorescence intensity (MFI) of NKG2D surface expression was reduced by ~90% on CD8+ T cells and by ~85% on NK cells in the patient sample and the frequency of CD8 T cells and NK cells expressing NKG2D on the surface was reduced by >50% and ~85% respectively, compared to the control sample assessed in parallel (**Figure 1B**). As an additional control, we evaluated CD5 expression on the patient’s T cells and as expected, its expression was preserved since glycosylation is not a pre-requisite for CD5 surface expression (**Figure 1B**; lower row). As has been reported previously, we also observed an increased frequency of circulating TCRαβ+ “double-negative” T cells (CD3+CD4-CD8-) in the patient’s blood (4.5% of CD3+ T cells; normal range <2.6%); (**Figure 1C**; upper-row) (3, 10). Additionally, consistent with published studies, we also observed an increased frequency of circulating CD5+ “innate-like” B1-B cells and elevated CD5 MFI on the patient’s B cells (**Figure 1C**; lower-row) (11). Specifically, the MFI of CD5 expression on B cells was almost two-fold higher than that in the control samples and the frequency of the patient’s CD19+ B cells co-expressing CD5 was also increased.

MAGT1 is composed of a thioredoxidin-like domain followed by 4 transmembrane segments (12). The mature MAGT1 protein is produced by removal of the signal peptide to leave residue Gln62 in the starting position and is integrated as a key subunit into the larger human oligosaccharyltransferase complex, OST-B, which catalyzes N-linked glycosylation on select target proteins. An OST-B structure has been determined by cryoEM; however, only the final 3 transmembrane helices of MAGT1 were resolved (13). An AlphaFold3-generated model of the full-length MAGT1 was overlaid onto the MAGT1 segments and fit into the OST-B structure making contact only with the STT3B protein subunit (**Figure 1D**) (5). The substrate for glycosylation by OST-B binds between MAGT1’s thioredoxin-like domain and a STT3B domain both exposed to the ER lumen. A CXXC motif located on the thioredoxidin-like domain is associated with MAGT1’s specialized enzymatic redox activity. The MAGT1 Ser194Phefs*3 variant introduces a stop codon near the end of the thioredoxin-like domain and lacks the entire transmembrane region. The missing transmembrane region is likely crucial for docking MAGT1 to STT3B in the OST-B complex.

Several observations are consistent and support the role of transmembrane helices 2–4 as an anchor for relative positioning of the thioredoxidin-like domain relative to STT3B. MAG-T1 density is not present in the OST-B cryoEM structure before transmembrane helix 2 (**Figure 1E**). Structures of OST-B fit MAGT1 transmembrane helices snuggly against STT3B, while in OST-A these proteins are replaced by DC2 and STT3A (13). Swapping MAGT1 into the OST-A structure or DC2 into the OST-B structure creates extensive steric clashes suggesting the high degree of shape complementarity is responsible for a tight, selective interaction. We explored the importance of the intermolecular transmembrane interaction further by analyzing the MAGT1 structure within the context of an AlphaFold MAGT1-STT3B complex. Relative to STT3B in the complex, AlphaFold3 positions MAGT1’s transmembrane helices 2-4, with higher confidence and lower structural variability than other regions in MAGT1 (**Figure 1F**). For the MAGT1 Ser194Phefs*3 variant which lacks all 4 transmembrane helices, there are no regions of the protein that show high positional confidence or low structural variability within a co-folded STT3B complex.

Without the transmembrane segments for anchoring and interaction specificity, it is unlikely for the MAGT1 Ser194Phefs*3 variant to be appropriately positioned within OST-B to help mediate enzymatic activity, even in the scenario of no impact on protein expression levels. The OST-B complex primarily targets proteins that are missed by OST-A including numerous membrane proteins involved in immune regulation and for the MAGT1 Ser194Phefs*3 variant patient, those targets would be mis-glycosylated or not glycosylated. TUSC3 is homologous to MAGT1 and is capable of rescuing N-glycosylation when MAGT1 is absent; however, many immune cells including CD8^+^ T cells and NK cells do not express TUSC3 (12). As mentioned previously, decreased surface expression of NKG2D due to impaired glycosylation by OST-B has been linked to “X-linked MAGT1 deficiency with increased susceptibility to EBV-infection and N-linked glycosylation” (XMEN) disease (3). This protein structure function analysis matches the diagnostic assessment of the patient and supports a molecular mechanism of a dysfunctional OST-B complex as the root cause of immunological impairment.

The patient’s asthma was initially treated with inhaled corticosteroids and omalizumab (an anti-IgE biologic therapy). In the last 18 months, the patient was transitioned from omalizumab to dupilumab, (an anti-IL-4Rα biologic that disrupts both IL-4 and IL-13 mediated signaling) (14). With this therapy, his asthma is better controlled and the total IgE level has begun to decline. His allergic rhinitis has been managed using over-the-counter medication and subcutaneous allergen immunotherapy. Given the patient’s infection history, sub-optimal response to the pneumococcal vaccine and persistently decreased IgG levels, immunoglobulin replacement therapy was initiated at the age of 6y when the patient first presented at our clinic. The patient’s warts and molluscum persist despite treatment with topical Cidofovir 3%. As XMEN patients display a heightened susceptibility to EBV infection and EBV-associated malignancies, the patient undergoes biannual assessments for EBV exposure in the cancer predisposition clinic. EBV DNA testing is performed using a clinically-validated quantitative amplification assay with analyte-specific reagents from Roche (Indianapolis, IN) on the company’s LightCycler instruments. This assay utilizes whole blood samples and the patient has consistently tested negative thus far (limit of detection is ≥2 copies/μL). Longitudinal complete metabolic profiling (CMP) did reveal asymptomatic elevated liver enzymes in the past, but these levels are now within normal limits with the exception of serum alanine aminotransferase (ALT) (latest ALT value: 88 IU/L (Reference Range: 5–50 IU/L). As is the case with most XMEN patients, our patient does not display clinically overt evidence of hepatic dysfunction, but we have incorporated evaluation by the gastroenterology service into the patient management plan (3, 10). There is no clinical evidence of platelet dysfunction or splenomegaly (based on abdominal CT).

## Discussion

We present an unexpected diagnosis of MAGT1 deficiency in a patient without complications from EBV infection but with symptoms predominated by significant atopy, persistent skin issues (warts and molluscum) and recurrent infections in the setting of hypogammaglobulinemia. To the best of our knowledge, neither atopy nor elevated IgE have been previously described in the setting of confirmed MAG-T1 deficiency. Intriguingly, autosomal recessive (AR) phosphoglucomutase 3 (*PGM3*) deficiency (another congenital disorder of glycosylation) that compromises N- as well as O-linked glycosylation is associated with atopy, immune deficiency, autoimmunity and neurocognitive impairment (15). IgE is a heavily glycosylated molecule with seven potential N-glycosylation sites residing within the conserved portion of the heavy chain, however, the role glycosylation plays in regulating IgE function is currently not well understood (16). We speculate that the MAG-T1 deficiency associated aberrant glycosylation might also impact the function of IgG4 antibodies that are known to regulate IgE-mediated responses (17, 18). However, another confounding variable in this regard is the family history of atopy, hence, at this stage, it is difficult to conclusively determine the extent to which the underlying MAGT1 defect is contributing to the patient’s atopy. We acknowledge that this as well as a lack of TH1/TH2 analysis is a limitation of our current study and we will attempt to elucidate this association in a follow-up report. Nevertheless, from a clinical management standpoint, it might be prudent to consider an underlying IEI as a potential diagnosis in patients presenting with significant atopy (that responds sub-optimally to standard medical therapy) if the clinical picture also includes other features that raise the suspicion for an underlying IEI.

Based on our survey of the literature, sixty-one MAGT1 deficiency patients have been reported thus far, with the disease showing complete penetrance but variable expressivity (19). The disease phenotypes reported continue to expand with initial studies focused on the morbidity and mortality associated with EBV-induced lymphomas (20). It is noteworthy, however, that a recent case report has expanded the spectrum of EBV-driven morbidity in XMEN disease by highlighting a case with EBV-mediated T-lymphocytic proliferative myositis without lymphoma development (21).

Extending beyond immune dysregulation, neuro cognitive impairment as well as neuropsychiatric symptoms have also been identified in the setting of MAGT1 deficiency (22, 23). However, as opposed to AR-PGM3 deficiency where neurocognitive impairment is frequently manifested, neurological changes are not identified in all MAGT1 deficiency patients (15).

B cell deficiency is not a classic finding in patients with MAGT1 deficiency (B cells can be frequently increased), but hypogammaglobulinemia is frequently identified (3, 10, 24). Notably, a 1999 study reported an expanded population of circulating CD5+ B cells (B1-B cells) in patients with selective anti-polysaccharide antibody deficiencies (now known as Specific Antibody Deficiency) (25). CD5+ B cells produce natural Abs and are considered providers of rapid first line immunity and are also implicated in the development of autoimmunity and leukemic B cell transformation (26–30). These unconventional B cells are present in large numbers in the fetal spleen and umbilical cord blood but usually decline to 10% by adulthood (28). Intriguingly, re-testing of one of the patients from the aforementioned 1999 study, who later presented with recurrent infections and autoimmunity, revealed him to be MAGT1 deficient (11). Our patient too did have a poor response to the polysaccharide vaccine challenge in the setting of expanded CD5+ (B1) B cells (**Figure 1C**; lower row). Hence, B1-B cell expansion might be part of the XMEN disease phenotype and could potentially be included in the laboratory work up to diagnose patients with XMEN disease.

Overall, the patient is doing satisfactorily with occasional asthma flares related to viral upper respiratory tract infections and has had two recent episodes of presumed bacterial pneumonia. With the transition to dupilumab therapy, his asthma has significantly improved and the total IgE level is down-trending [latest two assessments: 935 KU/L and most recently 573 KU/L (RR: < 176 KU/L)]. We speculate these changes are due to a combination of being off omalizumab, specifically as it increases free IgE levels, and from improvement of his atopic disease with dupilumab, which can both lend to clinical improvement and further lower total IgE. As biologics that target TH2 cytokines (such as dupilumab) have been reported to tilt the CD4 T cell repertoire balance towards TH1 and TH17 responses resulting in adverse effects including ocular surface disease, psoriasiform manifestations, facial redness and perhaps musculoskeletal inflammation, we will closely monitor the patient for these adverse effects (31). Furthermore, this dupilumab-induced TH2→TH1 shift could potentially add a confounding variable related to the proposed TH1/TH2 phenotypic assessment we propose to resolve the patient’s atopic phenotype.

We continue to monitor serum EBV by PCR regularly: testing remains negative to date. In the event the patient does get infected with EBV, this dupilumab induced TH1 shift might actually not be additionally detrimental in terms of controlling EBV-induced lymphoproliferation. Although it is well known that CD8 T cells are the predominant cell type involved in keeping EBV infection under check, there is well established evidence that cytotoxic (TH1) CD4 T cells are generated in the setting of primary EBV infection as well and that these CD4 T cells are capable of eliminating EBV-infected B cells *in vitro* (32–35). Furthermore, adoptive transfer of EBV-specific CD4 T cells in patients with EBV-associated post-transplant lymphoproliferative disease (PTLD) can induce sustained disease remission by virtue of both their ability to directly kill EBV-infected B cells and also generate immunostimulatory cytokines that facilitate killing of the target cells by EBV-specific CD8 T cells (36). Moreover, and to the best of our knowledge, CD4 T cells generally do not express NKG2D and are therefore not reliant on its expression to execute their effector function. Hence, dupilumab induced TH1 shift in the setting of MAGT1 deficiency (with concomitant loss of NKG2D expression) is unlikely to compromise CD4 T cell cytotoxic effector function against EBV-infected targets, although EBV immunosurveillance will still be sub-optimal due to the lack of CD8 T cell (and NK cell) associated NKG2D expression (37).

This case highlights the importance of taking an expansive history and referring patients to an immunology specialist as well as utilizing esoteric laboratory testing early in the immune work up, even in patients with atypical clinical presentations. This study also underscores the value of adopting an integrated cross-disciplinary approach that includes incorporating sophisticated analytic tools, such as AlphaFold, to complement conventional analytic platforms such as flow cytometry and genomics, in an effort to better characterize and define the underlying molecular defect.

## Data Availability

The original contributions presented in the study are included in the article/supplementary material, further inquiries can be directed to the corresponding author/s.
